# Associations of dental anxiety, depression, and general anxiety: A structural equation modeling study in the Northern Finland Birth Cohort 1986

**DOI:** 10.1111/eos.70062

**Published:** 2026-01-07

**Authors:** Mika Kajita, Priyanka Choudhary, Vesa Pohjola, Gerald Humphris, Jouko Miettunen, Satu Lahti

**Affiliations:** ^1^ Department of Community Dentistry University of Turku Turku Finland; ^2^ Research Unit of Clinical Medicine University of Oulu Oulu Finland; ^3^ Research Unit of Population Health University of Oulu Oulu Finland; ^4^ School of Medicine University of St Andrews St Andrews UK; ^5^ Medical Research Center Oulu Oulu University Hospital and University of Oulu Oulu Finland

**Keywords:** cohort studies, mental health, oral health, surveys and questionnaires

## Abstract

We aimed to estimate the associations between anticipatory and treatment‐related dental anxiety and depression and general anxiety at the latent level. This cross‐sectional study analyzed data from 3320 adults aged 33–35 years in the Northern Finland Birth Cohort 1986. Dental anxiety was measured with the Modified Dental Anxiety Scale and general anxiety and depression with the Hopkins Symptom Checklist‐25. Confirmatory factor analyses supported a two‐factor model with a residual correlation for dental anxiety (comparative fit index [CFI] = 0.999, root mean square error of approximation [RMSEA] = 0.038). Structural equation modeling was used to estimate primary latent correlations between anticipatory dental anxiety, treatment‐related dental anxiety, depression, and general anxiety. Secondary models adjusted for sex, education, and smoking. Depression and general anxiety correlated strongly (*r* = 0.72). Both anticipatory and treatment‐related dental anxiety showed modest associations with general anxiety (*r* = 0.16–0.18), whereas associations with depression were weaker and attenuated after adjustment. The two dental anxiety constructs were strongly interrelated (*r* = 0.85). Female sex, lower education, and smoking predicted higher dental anxiety. These findings support the distinctiveness of the two constructs of dental anxiety from depression and general anxiety, though partly overlapping with the latter. Future research should further clarify their developmental pathways and shared mechanisms.

## INTRODUCTION

Dental anxiety is a common global public health problem causing avoidance of dental treatment [1], which can further lead to a deterioration of oral health and poor oral health‐related quality of life [2, 3]. Given its impact on both individual well‐being and healthcare systems, understanding the nature and correlates of dental anxiety remains an important research priority. In the psychological literature, dental anxiety is typically distinguished from related constructs such as *dental fear*—specific emotional responses to threatening stimuli—and *dental phobia*—a severe clinical condition requiring psychiatric diagnosis [4, 5, 6, 7]. Dental anxiety is frequently assessed at the population level [8], reflecting its relevance as a construct that captures both anticipatory worry and treatment‐related distress. Public health research therefore has much to offer our understanding of these common and important psychological phenomena.

Beyond its clinical relevance, *dental anxiety* can be situated within broader psychological frameworks of fear and phobia. In dentistry, three related but distinct constructs are often mentioned: *dental fear* refers to immediate emotional responses to threatening dental stimuli (e.g., drilling, injection), *dental anxiety* denotes a more generalized apprehension and anticipation of dental treatment [4, 5, 6], whereas *dental phobia* represents a severe, persistent condition that meets diagnostic criteria for specific phobia in the Diagnostic and Statistical Manual of Mental Disorders [7], typically accompanied by marked avoidance. These distinctions align with general psychological models of fear and anxiety. Classical conditioning emphasizes the role of painful or aversive dental experiences in the development of dental fear [9], whereas cognitive models highlight the contribution of anticipatory processes and catastrophic thinking [10, 11]. The fear‐avoidance model [12], widely applied in pain and phobia research, also provides a useful framework: anxious anticipation leads to avoidance of dental care, reinforcing fear and maintaining the cycle of poor oral health [13]. Thus, dental anxiety shares common mechanisms with other strong fears and phobias, while also exhibiting unique clinical relevance in oral health contexts.

The five‐item Modified Dental Anxiety Scale (MDAS) was originally conceived as unidimensional [14], but accumulating psychometric findings indicate that it can identify two theoretically relevant components of dental anxiety: anticipatory (Items 1–2) and treatment‐related (Items 3–5) [15, 16, 17, 18, 19, 20]. Conceptually, anticipatory dental anxiety reflects future‐oriented, cognitive apprehension (e.g., anticipation and waiting), whereas treatment‐related dental anxiety reflects in‐procedure, somatic/conditioned fear to invasive stimuli (drilling, injection) [20]. Analogous dimensions—anticipatory/avoidance aspects versus stimulus‐specific fear—have also been noted in other dental fear measures such as the Dental Fear Survey [21]. Additional evidence suggests differential associations with external constructs: for example, treatment‐related dental anxiety has more firm associations to pain threshold in men than in women [22], whereas oral‐health‐related quality of life appears to be more strongly associated with anticipatory than treatment‐related dental anxiety [20]. Although these findings suggest some discriminant validity of the two‐factor structure, evidence regarding their associations with broader psychological constructs such as depression and general anxiety have been less consistent.

A systematic review has shown that both depression and general anxiety are more common among individuals with dental anxiety than in those without [23], and population‐based studies not included in the review have reported similar results [24, 25]. The systematic review also noted that the strength of these associations has varied across studies, partly due to differences in analytic approaches and study populations, which limits the generalizability of findings [23]. Besides the differences in the strength of the association, two of the existing studies assessing the relationships between different constructs of dental anxiety with depression and general anxiety had limitations; for example, the participants were university students or mothers and fathers expecting a baby [18, 26]. Moreover, of the studies evaluated, only one modeled the simultaneous association of depression and general anxiety with two factors of dental anxiety [18].

In addition, prior work has mainly relied on sum scores, which can be affected by measurement error and may obscure true associations. In contrast, latent variable modeling allows measurement error to be accounted for, providing more accurate estimates of the associations between psychological constructs [27, 28]. To our knowledge, no study has yet applied this approach to assess the associations of dental anxiety with both general anxiety and depression. This methodological gap may partly explain the inconsistencies in prior findings regarding associations of anticipatory dental anxiety and treatment‐related dental anxiety with depression and general anxiety.

Taken together, these gaps highlight the need for population‐based analyses that model latent constructs of dental anxiety, general anxiety, and depression simultaneously. Therefore, this study aimed, first, to evaluate the two‐factor structure of the MDAS in a general population and, second, to evaluate whether anticipatory and treatment‐related dental anxiety co‐occur with depressive and general anxiety symptoms, by modeling correlations among the latent constructs in the Northern Finland Birth Cohort (NFBC) 1986 study. We hypothesized that the MDAS would show a two‐factor structure and that both constructs would be modestly associated with depression and general anxiety.

## MATERIAL AND METHODS

### Study design and sample

This cross‐sectional population‐based study utilized data collected in the NFBC1986, covering the two northernmost provinces of Finland. The original cohort consisted of 9432 children (99% of all children born alive) whose expected date of birth fell between July 1985 and June 1986. More details of the NFBC1986 data collection and cohort design can be found in previous reports [29, 30, 31]. The data used in this study were collected when the participants were between 33 and 35 years old.

As part of the follow‐up, postal questionnaires were sent to all eligible cohort members (*N* = 8895), and a health examination was offered to those living in Oulu and surrounding areas (within a 250 km radius, *N* = 5740). Data collection took place between May 2019 and December 2020. In total, 3322 (37.4%) participants responded to Questionnaire I, which included the Hopkins Symptom Checklist‐25 (HSCL‐25), and 2869 (32.3%) responded to Questionnaire II, which included the MDAS.

The Ethics Committee of the Northern Ostrobothnia Hospital District approved the research (108/2017). All participants provided written informed consent. The inclusion criteria were participants who answered at least one item in either the MDAS or the HSCL. No exclusion criteria were set.

There is no universally accepted method for determining the optimal sample size for latent variable models. However, previous recommendations suggest that a minimum of 100–200 cases is required to perform structural equation modeling (SEM), with at least 100 cases per group for multigroup SEM [32]. Moreover, simulation studies have shown that complex models may require larger sample sizes to ensure adequate power and solution stability [33]. Our analyses examined correlations among four latent constructs and regression models adjusting for sex, education, and smoking. The final analytic sample of 3320 participants was therefore considered more than sufficient to ensure robust and interpretable results.

### Measures

All the measures included in the study were self‐reported using questionnaires.

Dental anxiety was measured using the MDAS, a valid and reliable five‐item instrument for self‐rating dental anxiety translated into Finnish [14, 34, 35]. The questions in the MDAS were: (Item 1) if you went to your dentist for treatment tomorrow, how would you feel; (Item 2) if you were sitting in the waiting room (waiting for treatment), how would you feel; (Item 3) if you were about to have a tooth drilled, how would you feel; (Item 4) if you were about to have your teeth scaled and polished, how would you feel; and (Item 5) if you were about to have a local anesthetic injection in your gum, above an upper back tooth, how would you feel? Each item had five response options, ranging from 1 (not anxious) to 5 (extremely anxious), with a total sum score range of 5–25. The cutoff point for high dental anxiety is 19, and the cutoff point for low dental anxiety is 10 [36]. The two constructs of dental anxiety were calculated: anticipatory dental anxiety (Items 1 and 2; score range = 2–10) and treatment‐related dental anxiety (Items 3–5; score range = 3–15) [15], and we employed the same structure in our analysis.

General anxiety and depression were assessed using the Finnish version of the HSCL‐25 [37, 38], a commonly used screening instrument for symptoms of anxiety (10 items) and depression (15 items) [39]. Responses were recorded on a 4‐point Likert scale ranging from 1 (not at all) to 4 (extremely). The depression subscale of the HSCL‐25 has also shown predictive value for future hospitalizations due to major depression in population‐based studies [40]. HSCL‐25 has shown varying factor structures across populations [38, 41, 42, 43, 44, 45, 46]. A three‐factor structure (anxiety, depression, and distress) was observed in the NFBC1966 previously [46].

Of the possible confounders, sex assigned at birth, education, and smoking, which have been previously associated with dental anxiety and mental health, were included [47, 48, 49, 50, 51, 52]. Smoking was categorized into three categories: 1 = nonsmoker, 2 = former smoker, and 3 = current smoker. Education was classified into two categories: 1 = low (from comprehensive school to polytechnic or bachelor's degree) and 2 = high (master's degree or higher).

### Statistical analysis

We employed SEM to examine associations between dental anxiety, depression, and general anxiety. Within this framework, confirmatory factor analyses (CFA) were first conducted to evaluate the factor structures of the MDAS and the HSCL‐25 in this cohort. Measurement invariance across sex was examined using multiple‐group CFA for both instruments. As HSCL did not achieve the required level of measurement invariance, multi‐group SEM by sex was not performed.

After establishing the measurement models, correlations among the latent constructs of anticipatory dental anxiety, treatment‐related dental anxiety, depression, and general anxiety were estimated. In additional analyses, latent regression models were performed to assess these associations while adjusting for sex, education, and smoking. Due to non‐normal distributions observed in the MDAS and HSCL items (i.e., skewed response patterns), SEM was performed using the robust maximum likelihood estimator (MLR). We report standardized factor loadings [53].

The model fit was assessed using the following indices: chi‐square/degree of freedom ratio (*χ* ^2^/df), comparative fit index (CFI), Tucker–Lewis index (TLI), root mean square error of approximation (RMSEA), standardized root mean square residual (SRMR), Akaike information criterion (AIC), and Bayesian information criterion (BIC). A CFI and TLI above 0.90 and RMSEA below 0.08 were taken to indicate an acceptable fit, whereas a CFI and TLI > 0.95 and RMSEA < 0.06 were considered indicators of a good fit. An SRMR < 0.08 was also regarded as indicative of a good fit. For AIC and BIC, lower values indicate better model fit when comparing alternative models [53].

As a preliminary step in evaluating the handling of missing data, Little's test was conducted to examine the missing completely at random (MCAR) assumption [54]. Because the MCAR assumption was rejected, full information maximum likelihood (FIML) estimation under the more general missing at random (MAR) assumption was adopted. FIML yields unbiased and efficient estimates relative to listwise deletion when MAR holds. This approach also allows for the inclusion of participants with partially missing data and is considered appropriate when missingness is conditionally dependent on observed variables. To evaluate the robustness of this approach, we additionally performed sensitivity analyses restricting the sample to participants meeting predefined coverage thresholds within each construct based on the final retained items (anticipatory dental anxiety 2/2 items; treatment‐related dental anxiety ≥2/3 items; HSCL‐depression ≥7/9 items; HSCL‐anxiety ≥2/3 items).

As another sensitivity analysis using conventional methods, we restricted the sample to participants with complete responses on all items of the MDAS, HSCL‐10 anxiety subscale, and HSCL‐15 depression subscale. For these participants, we computed total scores for each scale and estimated Pearson correlations between anticipatory dental anxiety, treatment dental anxiety, general anxiety, and depression. The sensitivity analyses based on sum scores were conducted using spss statistics, version 30.0.0.0 (IBM). SEM was conducted using r version 4.0.0 (R Foundation for Statistical Computing) and Package “lavaan” [55]. All tests were conducted at a significance level of 0.05.

We followed the STROBE guidelines for cross‐sectional studies; a completed checklist is provided in the Supporting Information.

## RESULTS

### Sample characteristics and missing data

Participants with missing values on all HSCL items and allMDAS items were excluded. Only cases with at least one non‐missing response across the 30 items (HSCL1–25 and MDAS1–5) were retained for analysis (*N* = 3320). MDAS items had approximately 14% missing values, and HSCL items had 5%–10%. Little's MCAR test was significant (*χ* ^2^(1505) = 2077.50, *p* < 0.001), indicating that data were not missing completely at random (MCAR). Therefore, FIML estimation was used under the MAR assumption.

To evaluate the adequacy of item‐level response patterns, we also identified the proportion of participants with sufficient valid responses within each construct: anticipatory dental anxiety (2 items), 2844 (85.7%); treatment‐related dental anxiety (≥2 of 3 items), 2855 (86.0%); HSCL‐depression (≥7 of 9 items), 3303 (99.5%); and HSCL‐anxiety (≥2 of 3 items), 3304 (99.5%). These results support the appropriateness of applying FIML to the full sample in subsequent analyses.

The sample characteristics are shown in Table 1. Between the sexes, current smoking rates were higher among males (41.2%) than among females (26.4%). Descriptive statistics for individual items of the MDAS and HSCL‐25 are shown in Table S1.

**TABLE 1:** Educational background and smoking status by gender.

	All *N* = 3320		Male *N* = 1258 (37.9%)		Female *N* = 2062 (62.1%)	
	*N*	%	*N*	%	*N*	%
Education						
Low	2339	70.5	914	72.7	1425	69.1
High	959	28.9	337	26.8	622	30.2
Missing	22	0.7	7	0.6	15	0.7
Smoking						
Nonsmoker	1128	34.0	370	29.4	758	36.8
Former smoker	1111	33.5	364	28.9	747	36.2
Current smoker	1062	32.0	518	41.2	544	26.4
Missing	19	0.6	6	0.5	13	0.6

*Note*: Low: from comprehensive school to polytechnics/bachelor's degree; high (master's degree or higher).

### Factor structure and reliability of the MDAS

To evaluate the dimensionality of the MDAS, we compared a one‐factor solution with the hypothesized two‐factor structure. The one‐factor model showed poor fit (e.g., CFI < 0.90, RMSEA > 0.30), whereas the hypothesized two‐factor solution demonstrated substantially better fit. A model allowing residual covariance between Items 3 and 5 yielded excellent indices (CFI ≈ 1.00, RMSEA < 0.05), replicating previous findings (Table 2). These results clearly support the superiority of the two‐factor solution over the unidimensional model. Internal consistency was high for both subscales (anticipatory: *α* = 0.94; treatment‐related: *α* = 0.88) as well as for the total scale (*α* = 0.92), indicating that the MDAS and its subcomponents were reliable measures in this population. Measurement invariance across sex was further examined (Table S2). Configural and metric invariance were supported, whereas scalar invariance was not established, indicating that factor loadings were comparable across groups, but latent mean comparisons could not be conducted.

**TABLE 2:** Model fit indices for confirmatory factor analyses of the MDAS (*N* = 2855).

Model	*χ* ^2^ (Scaled)	df	CFI (Robust)	TLI (Robust)	RMSEA (Robust)	SRMR	AIC	BIC
One‐factor model	1086	5	0.884	0.769	0.308	0.056	32638	32727
Two‐factor model	37	4	0.996	0.99	0.064	0.013	31344	31439
Two‐factor + residual covariance (mdas3–mdas5)	12	3	0.999	0.997	0.038	0.005	31310	31412

*Note*: All fit indices are reported in their robust (Yuan–Bentler) versions based on the MLR estimator. SRMR, AIC, and BIC are unaffected by robust corrections.

Abbreviations: AIC, Akaike information criterion; BIC, Bayesian information criterion; CFI, comparative fit index; MDAS, modified dental anxiety scale; RMSEA, root mean square error of approximation; SRMR, standardized root mean square residual; TLI, Tucker–Lewis index.

### Factor structure and reliability of the HSCL

The original factor structure of the HSCL‐25 did not replicate well in this cohort. After excluding items with weak correlations (<0.30), a 12‐item two‐factor model (9 items for depression and 3 items for anxiety) showed acceptable fit and was used in subsequent analyses. Measurement invariance across sex was also examined. Configural and metric invariance were supported, whereas scalar invariance was not established, indicating that latent mean comparisons between men and women were not appropriate. Details of the item selection and model fit indices are provided in Text S1, Tables S3–S5, and Figure S1.

### Latent variable correlations between dental anxiety, anxiety, and depression

**FIGURE 1:**
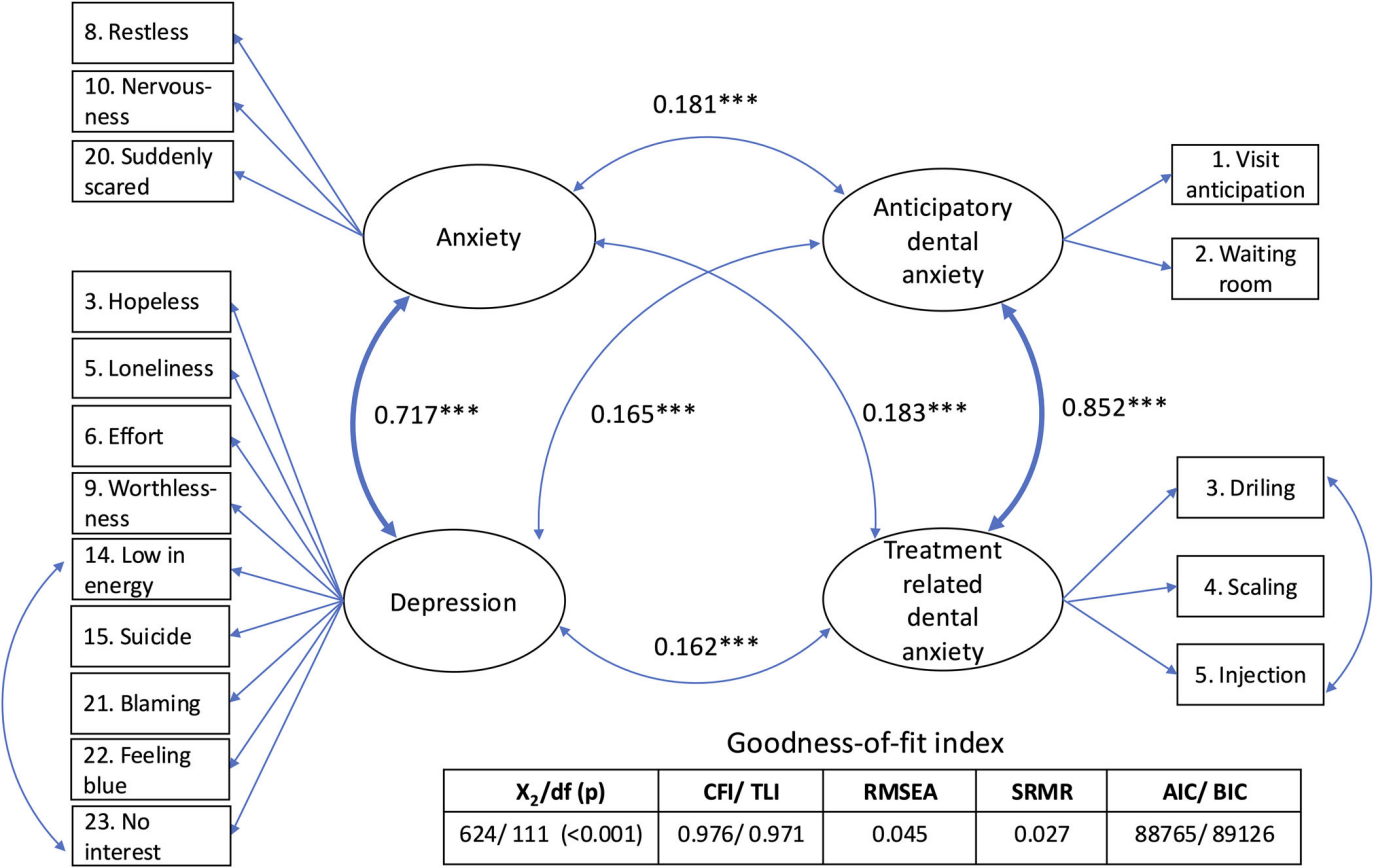
Structural equation model of associations between dental anxiety, general anxiety, and depression. The figure illustrates latent correlations among anticipatory dental anxiety, treatment‐related dental anxiety, general anxiety, and depression. Observed variables (boxes) load on their respective latent constructs (ellipses). Standardized latent correlations are displayed with significance levels (***p < 0.001). AIC, Akaike's information criterion; BIC, Bayesian information criterion; CFI, comparative fit index; RMSEA, root mean square error of approximation; SRMR, standardized root mean square residual; TLI, Tucker–Lewis index.

The structural model was specified with four latent constructs: anticipatory dental anxiety, treatment‐related dental anxiety, depression, and general anxiety. Figure 1 depicts the correlations between these latent variables. The two dental anxiety factors were strongly correlated, and depression and anxiety also showed a strong interrelationship. Depression and anxiety were modestly associated with anticipatory and treatment‐related dental anxiety in the unadjusted model (Figure 1).

In complete‐case analyses using traditional total scores (*n* = 2777–3210), anticipatory and treatment‐related dental anxiety correlated strongly with each other (*r* = 0.76) and weakly with general anxiety and depression (*r* = 0.17–0.19), whereas general anxiety and depression were strongly intercorrelated (*r* = 0.74) (Table S6). These patterns closely mirrored the latent correlations from the SEM with FIML (Figure 1), supporting the robustness of our main findings.

### Associations of depression and anxiety with dental anxiety, adjusted for covariates

In further analyses, we included sex, education, and smoking status as covariates in latent regression models. As shown in Table 3, the positive associations between general anxiety and both anticipatory and treatment‐related dental anxiety remained evident after adjustment, whereas the associations with depression attenuated and were no longer robust. Female sex, lower education, and smoking were independently associated with higher dental anxiety. As a sensitivity analysis, we restricted the sample to participants with sufficient valid responses within each construct (anticipatory dental anxiety: 2 items; treatment‐related dental anxiety: ≥2 of 3 items; HSCL‐depression: ≥7 of 9 items; HSCL‐anxiety: ≥2 of 3 items). The SEM results were materially unchanged (Table S7), suggesting that partial missingness did not substantially affect the findings.

**TABLE 3:** Associations of depression, anxiety, smoking, education, and gender with anticipatory and treatment‐related dental anxiety (*N* = 3320).

Dental anxiety dimensions	Correlates	*β* Unadjusted (95% CI)	*β* Adjusted (95% CI)
Anticipatory dental anxiety	Depression	0.07 (−0.00, 0.15)	0.07 (−0.01, 0.14)
	Anxiety	0.13 (0.05, 0.21)	0.12 (0.04, 0.20)
	Smoking	–	0.06 (0.02, 0.10)
	Education	–	−0.11 (−0.14, −0.07)
	Gender	–	0.16 (0.12, 0.19)
Treatment‐related dental anxiety	Depression	0.06 (−0.01, 0.14)	0.05 (−0.03, 0.12)
	Anxiety	0.14 (0.06, 0.22)	0.13 (0.05, 0.21)
	Smoking	–	0.06 (0.02, 0.10)
	Education	–	−0.04 (−0.08, −0.01)
	Gender	–	0.22 (0.18, 0.26)

*Note*: *β* = standardized regression coefficient; estimates from structural equation model with the robust maximum likelihood estimator (MLR) and full information maximum likelihood. Adjusted models included gender, smoking, and education; due to missing values in these covariates, the adjusted analyses were based on *N* = 3292. Robust fit: *χ* ^2^(111) = 623.98, CFI = 0.976, TLI = 0.971, RMSEA = 0.045 (90% CI 0.041–0.048), SRMR = 0.027.

## DISCUSSION

This population‐based study modeled correlations among latent constructs of anticipatory and treatment‐related dental anxiety, depression, and general anxiety. We observed a strong interrelation between general anxiety and depression, whereas the links between dental anxiety and these mental health dimensions were modest. After adjustment for sex, education, and smoking, the associations with general anxiety remained evident, whereas those with depression attenuated. Taken together, the results indicate that these four constructs co‐occur but are not equivalent; substantial unique variance remains for dental anxiety.

Given the cross‐sectional design, these patterns should be interpreted as concurrent associations rather than causal effects. In addition, estimates relied on FIML under the MAR assumption. Although Little's test rejected MCAR, the MAR assumption was supported by the availability of most relevant covariates, and sensitivity analyses restricted to participants with sufficient item coverage yielded materially similar results. Thus, our findings appear robust to potential biases due to missing data. Complementary analyses using sum‐score complete‐case data produced the same pattern of associations, further supporting the robustness of the findings and justifying the use of SEM with FIML as the main analytic approach.

Our latent model showed a strong relationship between depression and generalized anxiety but rather modest associations with anticipatory and treatment‐related dental anxiety; moreover, the links with depression attenuated after adjustment for sex, education, and smoking, whereas the associations with generalized anxiety persisted. This attenuation pattern suggests that the selected covariates (sex, education, and smoking) capture part of the shared variance between depression and dental anxiety. However, because smoking may also reflect the consequences of general anxiety rather than a simple confounder, the adjusted estimates should be interpreted with caution.

Interpreted within contemporary dimensional accounts of psychopathology, depression and generalized anxiety chiefly index the internalizing “distress” core, whereas situation‐bound fears map onto a “fear” subfactor [56, 57]. The observed pattern—especially the weak relation with the psychometrically robust depression factor—suggests that dental anxiety is not merely a manifestation of depressive symptomatology; its modest co‐occurrence with generalized anxiety is plausibly driven by shared physiological arousal and anticipatory threat. Thus, dental anxiety appears to overlap with, yet remain distinguishable from, the internalizing spectrum.

A key comparison can be made with our earlier work, which studied expectant mothers and their partners during pregnancy  [18]. In that study, generalized anxiety symptoms were associated with anticipatory dental anxiety but not treatment‐related dental anxiety, with a slightly stronger association in men. Depressive symptoms were associated with both anticipatory dental anxiety and treatment‐related dental anxiety, and the link between depression and anticipatory dental anxiety was stronger among women. In the present general adult cohort, however, generalized anxiety showed associations with both anticipatory dental anxiety and treatment‐related dental anxiety, with a stronger link for treatment‐related dental anxiety, whereas depressive symptoms were weakly related only to anticipatory dental anxiety, and the association attenuated after covariate adjustment. These contrasts suggest that pregnancy may amplify the connection between depression and dental anxiety, likely reflecting heightened emotional dysregulation and stress vulnerability during the perinatal period [58]. By contrast, in this general adult population, the associations appear more modest and uniform, underscoring the role of life context in shaping the links between mental health and dental anxiety.

We also assessed the factor structure of the HSCL‐25 in this cohort. The initially proposed three‐factor model (anxiety, depression, and distress), which fit well in an earlier Finnish birth cohort (NFBC1966) [18], did not replicate adequately in this younger cohort. The limited replication of the three‐factor model in this younger cohort supports the idea that factor structures can vary by age, time, or cultural context [37, 41, 42, 43, 44, 45, 46], especially for broad instruments like the HSCL. We therefore constructed a refined two‐factor solution (depression and anxiety) with a residual covariance, which showed an acceptable fit and was used in the analysis. The residual covariance was introduced where item content clearly overlaps—HSCL “low energy” with “no interest” (anhedonia/energy loss). Both items represent core features of depression, and prior symptom research has demonstrated that anhedonia and lack of energy are closely intertwined, making local dependence likely [59]. Thus, this residual correlation is interpreted as reflecting a substantive relationship rather than a mere statistical artifact. The three‐item latent factor for general anxiety, though empirically adequate for identification, may introduce some instability in SEM estimates. This limitation is acknowledged, and the observed consistency with sum‐score analyses supports the robustness of the findings.

Our study also confirmed the two‐factor structure of the MDAS in this cohort. Model comparisons supported a two‐factor solution over a one‐factor alternative, and adding a residual correlation between Items 3 (drilling) and 5 (injection) further improved fit, consistent with previous findings from a UK general population [20]. As both items concern invasive, pain‐related procedures, and the “injection” item was historically added to the Corah's Dental Anxiety Scale due to its comparable fear to drilling [60]. This specification reflects substantive item overlap rather than a post hoc statistical adjustment.

The excellent internal consistency of the overall MDAS and its two factors demonstrated high reliability. These results provide both theoretical and practical support for distinguishing anticipatory dental anxiety and treatment‐related dental anxiety, reinforcing their conceptual relevance. Nonetheless, the brevity of the subscales, especially the two‐item anticipatory dental anxiety, raises concerns about stability in SEM and indicates that path estimates involving these factors should be interpreted cautiously. Taken together, the MDAS can be considered useful as a single‐factor instrument, whereas the two‐factor structure offers superior fit and may be informative in contexts, such as clinical settings, where differentiation between anticipatory and treatment‐related anxiety is of interest.

Several strengths and limitations should be noted. A key strength of this study is the use of a large, population‐based cohort of adults. Instead of relying on simple sum scores, we applied SEM to examine correlations among latent variables, thereby accounting for measurement error. This approach reduces common method variance and provides more accurate estimates of the associations. To minimize potential bias, validated Finnish versions of the HSCL‐25 and MDAS were employed. A limitation is that all variables were self‐reported, introducing the possibility of reporting bias. Another limitation is the cross‐sectional design, which prevents causal inference. An additional limitation is the moderate response rate (37.4% for Questionnaire I and 32.3% for Questionnaire II), which is typical for long‐term cohort studies but may still limit generalizability, particularly as women were overrepresented (62%). The over‐representation of women (62%) suggests potential selection bias that could bias the associations of interest if participation was influenced by dental anxiety or related characteristics. Although we could not formally assess this due to lack of information on non‐participants, the consistency of sex‐adjusted patterns indicates that major distortion is unlikely. Adjustment was limited to sex, education, and smoking, leaving the possibility of residual confounding by unmeasured factors such as other socioeconomic or health‐related variables. The association between smoking and mental health is bidirectional in the literature; thus, smoking may not act as a simple confounder, and caution is needed when interpreting adjusted estimates. Furthermore, measurement invariance testing indicated that although configural and metric invariance were supported for both instruments, scalar invariance was not established. As a result, latent mean comparisons across sex could not be conducted, which limits the scope of between‐group inferences. Finally, as the cohort consisted of Finnish adults aged 33–35 years, so the findings may not generalize to other age groups or cultural contexts. Despite these limitations, this study makes two key contributions. First, it provides population‐based evidence supporting the two‐factor structure of the MDAS against a one‐factor alternative. Second, it is the first to model the concurrent associations of anticipatory and treatment‐related dental anxiety with depression and general anxiety at the latent level, highlighting both overlap and distinctiveness across these constructs.

In summary, dental anxiety shows modest co‐occurrence with depression and general anxiety, yet retains unique variance, supporting its conceptual distinctiveness within internalizing psychopathology. Future studies should include more comprehensive covariates, and longitudinal designs are needed to clarify causal pathways and developmental trajectories. Differentiating anticipatory and treatment‐related components may also provide a useful framework for developing tailored clinical or preventive approaches in the future. However, such implications should be considered tentative until replicated in longitudinal and clinical studies.

## AUTHOR CONTRIBUTIONS

**Conceptualization**: Satu Lahti, Mika Kajita, Vesa Pohjola. **Methodology**: Mika Kajita, Gerald Humphris, Satu Lahti, Jouko Miettunen. **Validation**: Mika Kajita, Priyanka Choudhary. **Formal analysis**: Mika Kajita, Priyanka Choudhary. **Data curation**: Mika Kajita, Priyanka Choudhary. **Funding acquisition**: Mika Kajita, Priyanka Choudhary. **Writing—original draft preparation**: Mika Kajita,Satu Lahti. **Writing—review and editing**: Mika Kajita, Satu Lahti, Vesa Pohjola, Priyanka Choudhary, Gerald Humphris, Jouko Miettunen.

## CONFLICT OF INTEREST STATEMENT

The authors declare no conflicts of interest.

## DISCLOSURE OF AI USE

The authors used ChatGPT (OpenAI, San Francisco, CA, USA) to assist with literature search and English language editing. All AI‐assisted outputs were carefully checked, revised, and verified by the authors, who take full responsibility for the final content.

## Supporting information



Supporting Information

## Data Availability

NFBC data are available from the University of Oulu, Infrastructure for Population Studies. Permission to use the data can be applied for research purposes via an electronic material request portal. In the use of data, the EU General Data Protection Regulation (679/2016) and the Finnish Data Protection Act are followed. To access the data, contact the NFBC project center (NFBCprojectcenter@oulu.fi) and visit the cohort website (www.oulu.fi/nfbc) for more information.
