# Supplementary material for: Associations of dental anxiety, depression, and general anxiety: A structural equation modeling study in the Northern Finland Birth Cohort 1986
Source: Eur J Oral Sci. 2026 Jan 7;134(2):e70062. doi: 10.1111/eos.70062 (PMC12976842; doi:10.1111/eos.70062)
Supplement: Supplementary file 1 — Supporting Information [file EOS-134-e70062-s001.docx]

SUPPORTING INFORMATION

**Associations of dental anxiety, depression, and general anxiety: a structural equation modeling study in the Northern Finland Birth Cohort 1986**

Mika Kajita,Priyanka Choudhary, Vesa Pohjola, Gerald Humphris, Jouko Miettunen, Satu Lahti

University of Turku, Finland

University of Oulu, Finland

University of St. Andrews, UK

Oulu University Hospital, Finland

Contents:

Table S1. Supplemental Table 1. Descriptive statistics for individual items of the MDAS and HSCL-25.

Table S2.

Text S1. HSCL-25 factor structure analyses

References

Table S3. Item–total correlations of HSCL-25 items used for item exclusion criteria (ρ < 0.30)

Table S4. Results of exploratory factor analysis (EFA) with maximum likelihood estimation and oblique rotation for HSCL-25 items

Figure S1. Confirmatory factor analysis results of the adopted two-factor model of the Hopkins Symptom Checklist (HSCL).

Table S5. Model fit indices for measurement invariance testing of the HSCL factor structure across gender.

Table S6. Sum-score correlation matrix for Anticipatory/Treatment Dental Anxiety, HSCL_ Anxiety (10 items), and HSCL_ Depression (15 items)

Table S7. Sensitivity analysis: Associations of depression, anxiety, smoking, education, and gender with anticipatory and treatment-related dental anxiety (N = 2829)

| **Table S1.** Descriptive statistics for individual items of the MDAS and HSCL-25. | | | | | |
| --- | --- | --- | --- | --- | --- |
|  | N | Mean | SD | Skewness | Kurtosis |
| MDAS |  |  |  |  |  |
| 1. Visit anticipation | 2855 | 1.7 | 1.0 | 1.7 | 2.3 |
| 2. Waiting room | 2844 | 1.8 | 1.1 | 1.4 | 1.2 |
| 3. Drilling | 2844 | 2.5 | 1.2 | 0.6 | -0.6 |
| 4. Scaling | 2846 | 2 | 1.1 | 1 | 0.6 |
| 5. Injection | 2847 | 2.4 | 1.1 | 0.6 | -0.4 |
| HSCL-25 |  |  |  |  |  |
| 1. Headaches | 3299 | 1.7 | 0.7 | 0.8 | 0.6 |
| 2. Sleep | 3299 | 1.6 | 0.7 | 1.2 | 1.1 |
| 3. Hopeless | 3296 | 1.4 | 0.7 | 1.8 | 3.1 |
| 4. Tense | 3302 | 1.9 | 0.8 | 0.6 | -0.1 |
| 5. Lonely | 3301 | 1.5 | 0.8 | 1.4 | 1.4 |
| 6. Effort | 3296 | 1.5 | 0.8 | 1.3 | 1.1 |
| 7. Terror | 3302 | 1.2 | 0.5 | 2.4 | 6.2 |
| 8. Restless | 3302 | 1.2 | 0.5 | 2.5 | 7 |
| 9. Worthlessness | 3302 | 1.4 | 0.7 | 1.8 | 2.8 |
| 10. Nervousness | 3302 | 1.5 | 0.7 | 1.1 | 0.9 |
| 11. Faintness | 3300 | 1.3 | 0.5 | 2.2 | 5.1 |
| 12. Worrying | 3301 | 1.8 | 0.7 | 0.7 | 0.4 |
| 13. Loss of sexual interest | 3299 | 1.5 | 0.8 | 1.5 | 1.8 |
| 14. Low in energy | 3299 | 1.8 | 0.8 | 0.9 | 0.4 |
| 15. Suicide | 3300 | 1.1 | 0.3 | 5.1 | 31.3 |
| 16. Trembling | 3301 | 1.1 | 0.3 | 5.3 | 34.2 |
| 17. Poor appetite | 3303 | 1.2 | 0.5 | 3.3 | 11.9 |
| 18. Crying | 3301 | 1.3 | 0.6 | 2 | 3.8 |
| 19. Trapped | 3295 | 1.1 | 0.4 | 4.1 | 18.7 |
| 20. Scared | 3297 | 1.2 | 0.4 | 3.1 | 10.6 |
| 21. Blaming | 3300 | 1.4 | 0.7 | 1.8 | 3.1 |
| 22. Feeling blue | 3299 | 1.5 | 0.7 | 1.3 | 1.5 |
| 23. No interest | 3299 | 1.5 | 0.7 | 1.3 | 1.4 |
| 24. Feeling fearful | 3300 | 1.2 | 0.5 | 2.8 | 8.2 |
| 25. Heart pounding | 3295 | 1.2 | 0.5 | 2.3 | 5.6 |
| Abbreviations: MDAS, Modified Dental Anxiety Scale; HSCL, Hopkins Symptom Checklist | | | | | |

**Table S2**. Model fit indices for measurement invariance testing of the MDAS factor structure across gender.

| Model | χ² | df | CFI | RMSEA | SRMR | AIC | Δχ² / Δdf (p) |
| --- | --- | --- | --- | --- | --- | --- | --- |
| Configural | 12.2 | 6 | 0.999 | 0.032 | 0.005 | 31024 | – |
| Metric | 17.4 | 9 | 0.999 | 0.030 | 0.014 | 31026 | 5.275 / 3 (p = .153) |
| Scalar | 41.6 | 12 | 0.997 | 0.048 | 0.021 | 31051 | 28.797 / 3 (p < .001) |
| Strict | 82.4 | 17 | 0.992 | 0.063 | 0.025 | 31105 | 36.596 / 5 (p < .001) |

Abbreviations: MDAS = Modified Dental Anxiety Scale; χ² = chi-square; df = degrees of freedom; CFI = Comparative Fit Index; RMSEA = Root Mean Square Error of Approximation; SRMR = Standardized Root Mean Square Residual; AIC = Akaike Information Criterion; Δχ² / Δdf = chi-square difference test.

Note. Configural and metric invariance were supported. However, scalar and strict invariance constraints significantly worsened model fit, indicating that full invariance could not be established across gender.

**Text S1**. the Hopkins Symptom Checklist (HSCL-25) factor structure analyses

Because the HSCL-25 has been reported to show varying factor structures across different populations, we first examined its dimensionality in the NFBC1986 sample. Initially, two subscales for anxiety and depression were assumed, but HSCL-25 has shown varying factor structures across populations [S1-S7]. A three-factor structure (anxiety, depression, and distress) was observed in the Northern Finland Birth Cohort 1966 (NFBC1966) previously [S7]. Thus, we first tested this three-factor model using confirmatory factor analysis (CFA) in the Northern Finland birth cohort 1986 (NFBC1986) cohort. However, the structure did not replicate well; Four items showed factor loadings below 0.50, and the three latent factors were very highly correlated (r > 0.84), raising concerns about multicollinearity when used as independent variables in SEM and the fit of the model was insufficient (χ² = 2840.85, df = 206, RMSEA = 0.062, CFI = 0.911, TLI = 0.900).

To obtain more distinct latent constructs of depression and anxiety, we excluded items that showed insufficient correlation (Spearman's ρ < 0.3) with other items, i.e., HSCL1 (headaches), HSCL2 (difficulties falling asleep)", HSCL11 (faintness, dizziness), HSCL16 (trembling) and HSCL17 (poor appetite) (Table S1).

Based on the theoretical structure of the HSCL-25, which was initially developed to assess symptoms of depression and anxiety, we conducted an exploratory factor analysis (EFA) with the number of factors fixed to two. The EFA was performed using maximum likelihood estimation and oblique rotation. Items with factor loadings above 0.50 (nine items for depression and three for anxiety) (Table S2) were retained for subsequent confirmatory factor analysis (CFA). The initial CFA of the 12-item model showed an acceptable fit (χ² = 548.58, df = 53, CFI = 0.950, RMSEA = 0.070, SRMR = 0.033). Based on modification indices, a residual covariance was added between HSCL14 (Low in energy) and HSCL23 (No interest), which improved the fit (χ² = 435.73, df = 52, CFI = 0.961, RMSEA = 0.062, SRMR = 0.030). This updated model (Figure S1) was used in the subsequent structural equation modeling (SEM).

**References:**

S1. Kuittinen S, García Velázquez R, Castaneda AE, Punamäki R-L, Rask S, Suvisaari J. Construct validity of the HSCL-25 and SCL-90-Somatization scales among Russian, Somali and Kurdish origin migrants in Finland. International Journal of Culture and Mental Health. 2017;10:1-18.

S2. Skogen JC, Øverland S, Smith ORF, Aarø LE. The factor structure of the Hopkins Symptoms Checklist (HSCL-25) in a student population: A cautionary tale. Scand J Public Health. 2017;45:357-65.

S3. Glaesmer H, Braehler E, Grande G, Hinz A, Petermann F, Romppel M. The German Version of the Hopkins Symptoms Checklist-25 (HSCL-25) --factorial structure, psychometric properties, and population-based norms. Compr Psychiatry. 2014;55:396-403.

S4. Ashaba S, Kakuhikire B, Vořechovská D, Perkins JM, Cooper-Vince CE, Maling S, et al. Reliability, Validity, and Factor Structure of the Hopkins Symptom Checklist-25: Population-Based Study of Persons Living with HIV in Rural Uganda. AIDS Behav. 2018;22:1467-74.

S5. Rodríguez-Barragán M, Fernández-San-Martín MI, Clavería-Fontán A, Aldecoa-Landesa S, Casajuana-Closas M, Llobera J, et al. Validation and Psychometric Properties of the Spanish Version of the Hopkins Symptom Checklist-25 Scale for Depression Detection in Primary Care. Int J Environ Res Public Health. 2021;18.

S6. Lundin A, Hallgren M, Forsell Y. The validity of the symptom checklist depression and anxiety subscales: A general population study in Sweden. J Affect Disord. 2015;183:247-52.

S7. Knuutila J, Lahti S, Sipilä K, Tolvanen M. Associations between pain-related temporomandibular disorders and dental anxiety at 46 years of age in the Northern Finland Birth Cohort 1966. Acta Odontol Scand. 2023;81:633-40.

| **Table S3**. Item–total correlations of HSCL-25 items used for item exclusion criteria (ρ < 0.30). | | | | | |
| --- | --- | --- | --- | --- | --- |
|  | HSCL1 | HSCL2 | HSCL11 | HSCL16 | HSCL17 |
| 1. Headaches | 1 | 0.165** | 0.246** | 0.120** | 0.122** |
| 2. Sleep | 0.165** | 1 | 0.126** | 0.142** | 0.179** |
| 3. Hopeless | 0.094** | 0.217** | 0.148** | 0.159** | 0.232** |
| 4. Tense | 0.250** | 0.243** | 0.218** | 0.165** | 0.216** |
| 5. Lonely | 0.129** | 0.233** | 0.170** | 0.151** | 0.204** |
| 6. Effort | 0.146** | 0.226** | 0.151** | 0.163** | 0.219** |
| 7. Terror | 0.147** | 0.211** | 0.219** | 0.194** | 0.244** |
| 8. Restless | 0.117** | 0.197** | 0.186** | 0.219** | 0.234** |
| 9. Worthlessness | 0.115** | 0.205** | 0.178** | 0.167** | 0.236** |
| 10. Nervousness | 0.157** | 0.265** | 0.188** | 0.191** | 0.248** |
| 11. Faintness | 0.246** | 0.126** | 1 | 0.192** | 0.161** |
| 12. Worrying | 0.161** | 0.212** | 0.214** | 0.142** | 0.217** |
| 13. Loss of sexual interest | 0.095** | 0.132** | 0.123** | 0.070** | 0.127** |
| 14. Low in energy | 0.187** | 0.237** | 0.194** | 0.162** | 0.238** |
| 15. Suicide | 0.041* | 0.146** | 0.116** | 0.131** | 0.139** |
| 16. Trembling | 0.120** | 0.142** | 0.192** | 1 | 0.224** |
| 17. Poor appetite | 0.122** | 0.179** | 0.161** | 0.224** | 1 |
| 18. Crying | 0.174** | 0.167** | 0.198** | 0.172** | 0.235** |
| 19. Trapped | 0.064** | 0.146** | 0.154** | 0.095** | 0.185** |
| 20. Scared | 0.111** | 0.173** | 0.176** | 0.179** | 0.232** |
| 21. Blaming | 0.109** | 0.201** | 0.167** | 0.146** | 0.209** |
| 22. Feeling blue | 0.157** | 0.253** | 0.174** | 0.184** | 0.243** |
| 23. No interest | 0.141** | 0.224** | 0.172** | 0.154** | 0.251** |
| 24. Feeling fearful | 0.138** | 0.213** | 0.208** | 0.200** | 0.248** |
| 25. Heart pounding | 0.168** | 0.192** | 0.268** | 0.221** | 0.182** |
| Abbreviations: HSCL, Hopkins Symptom Checklist | | | | | |
| Note. Spearman’s rho was used due to skewed item distributions. | | | | | |
| **p < .01 (two-tailed). | |  |  |  |  |

| **Table S4.** Results of exploratory factor analysis (EFA) with maximum likelihood estimation and oblique rotation for HSCL-25 items. | | |
| --- | --- | --- |
|  | Factor 1 | Factor 2 |
| 3. Hopeless | 0.790 | -0.069 |
| 4. Tense | 0.342 | 0.318 |
| 5. Lonely | 0.689 | -0.079 |
| 6. Effort | 0.632 | 0.103 |
| 7. Terror | 0.281 | 0.499 |
| 8. Restless | -0.078 | 0.725 |
| 9. Worthlessness | 0.773 | -0.041 |
| 10. Nervousness | 0.147 | 0.626 |
| 12. Worrying | 0.481 | 0.212 |
| 13. Loss of sexual interest | 0.419 | 0.003 |
| 14. Low in energy | 0.544 | 0.098 |
| 15. Suicide | 0.517 | -0.026 |
| 18. Crying | 0.423 | 0.136 |
| 19. Trapped | 0.277 | 0.247 |
| 20. Scared | -0.031 | 0.698 |
| 21. Blaming | 0.643 | 0.106 |
| 22. Feeling blue | 0.857 | -0.034 |
| 23. No interest | 0.692 | 0.027 |
| 24. Feeling fearful | 0.467 | 0.334 |
| 25. Heart pounding | 0.062 | 0.398 |

**
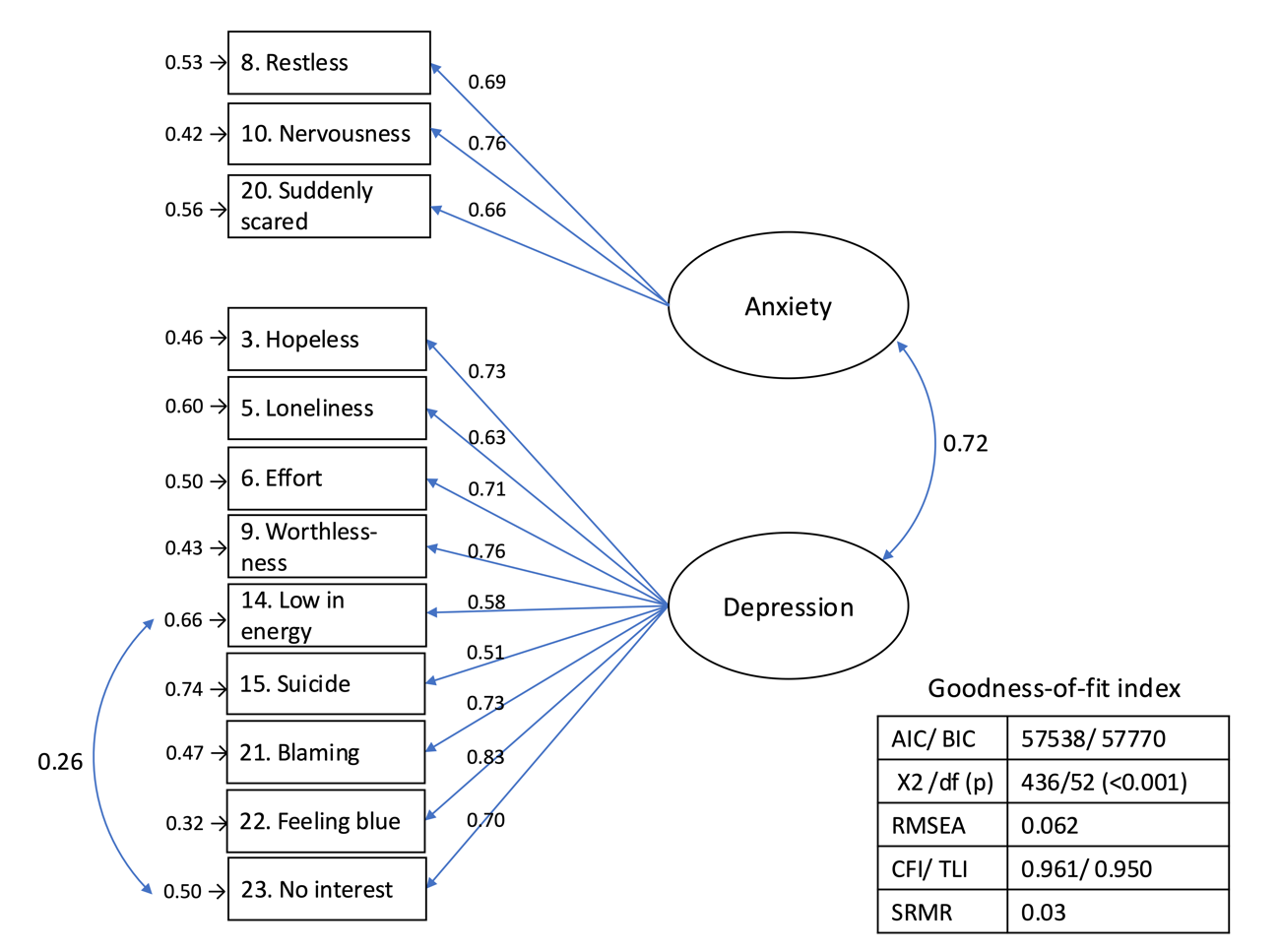
Figure S1**. Confirmatory factor analysis results of the adopted two-factor model of the Hopkins Symptom Checklist (HSCL). AIC, Akaike's information criterion; BIC, Bayesian information criterion; CFI, comparative fit index; TLI, Tucker–Lewis index; RMSEA, root mean square error of approximation; SRMR, standardized root mean square residual.

**Table S5**. Model fit indices for measurement invariance testing of the HSCL factor structure across gender.

| Model | χ² | df | CFI | RMSEA | SRMR | AIC | Δχ² / Δdf (p) |
| --- | --- | --- | --- | --- | --- | --- | --- |
| Configural | 521.8 | 104 | 0.959 | 0.064 | 0.033 | 57142 | – |
| Metric | 560.1 | 114 | 0.955 | 0.064 | 0.048 | 57212 | 40.7 / 10 (<.001) |
| Scalar | 668.9 | 124 | 0.947 | 0.067 | 0.050 | 57340 | 154.6 / 10 (<.001) |
| Strict | 733.2 | 136 | 0.938 | 0.069 | 0.060 | 57504 | 64.5 / 12 (<.001) |

*Note.* Configural invariance was supported. However, significant χ² difference tests indicated that metric, scalar, and strict invariance constraints substantially worsened model fit, suggesting that full invariance could not be established across gender.

**Table S6.** Sum-score correlation matrix for Anticipatory/Treatment Dental Anxiety, HSCL_ Anxiety (10 items), and HSCL_ Depression (15 items)

|  | Ant_da | Tt_da | HSCL_anx10 | HSCL_dep15 |
| --- | --- | --- | --- | --- |
| Ant_da | 1.00 | .760 (N=2817) | .193 (N=2800) | .178 (N=2777) |
| Tt_da | .760 (N=2817) | 1.00 | .187 (N=2783) | .171 (N=2762) |
| HSCL_anxiety | .193 (N=2800) | .187 (N=2783) | 1.00 | .736 (N=3210) |
| HSCL_depression | .178 (N=2777) | .171 (N=2762) | .736 (N=3210) | 1.00 |

*Notes.* Values are Pearson’s r; all p < .001 (two-tailed). N indicates pairwise complete cases for each correlation. Ant_da = anticipatory dental anxiety; Tt_da = treatment-related dental anxiety; HSCL= the Hopkins Symptom Checklist.

| **Table S7.** Sensitivity analysis: Associations of depression, anxiety, smoking, education, and gender with anticipatory and treatment-related dental anxiety (N = 2829) | | | |
| --- | --- | --- | --- |
| Dental anxiety dimensions | Correlates | β Unadjusted (95% CI) | β Adjusted (95% CI) |
| Anticipatory dental anxiety | Depression | 0.07 (−0.00, 0.15) | 0.07 (−0.01, 0.14) |
|  | Anxiety | 0.13 (0.05, 0.21) | 0.12 (0.04, 0.20) |
|  | Smoking | – | 0.06 (0.02, 0.10) |
|  | Education | – | −0.11 (−0.14, −0.07) |
|  | Gender | – | 0.15 (0.12, 0.19) |
| Treatment-related dental anxiety | Depression | 0.07 (−0.01, 0.14) | 0.05 (−0.03, 0.12) |
|  | Anxiety | 0.14 (0.05, 0.22) | 0.13 (0.04, 0.21) |
|  | Smoking | – | 0.06 (0.02, 0.10) |
|  | Education | – | −0.04 (−0.08, −0.01) |
|  | Gender | – | 0.22 (0.18, 0.25) |
| Notes. β = standardized regression coefficient; estimates from structural equation model with the robust maximum likelihood estimator (MLR) and full information maximum likelihood. Adjusted models included gender, smoking, and education; due to missing values in these covariates, the adjusted analyses were based on N = 2817. Robust model fit: χ²(111) = 529.68, CFI = 0.978, TLI = 0.973, RMSEA = 0.043 (90% CI 0.039–0.047), SRMR = 0.026. | | | |
